# The Quest for the Best: How TCR Affinity, Avidity, and Functional Avidity Affect TCR-Engineered T-Cell Antitumor Responses

**DOI:** 10.3390/cells9071720

**Published:** 2020-07-18

**Authors:** Diana Campillo-Davo, Donovan Flumens, Eva Lion

**Affiliations:** 1 Tumor Immunology Group, Laboratory of Experimental Hematology, Vaccine & Infectious Disease Institute (VAXINFECTIO), Faculty of Medicine and Health Sciences, University of Antwerp, 2610 Antwerp, Belgium; donovan.Flumens@student.uantwerpen.be (D.F.); eva.lion@uantwerpen.be (E.L.); 2 Center for Cell Therapy & Regenerative Medicine, Antwerp University Hospital, 2650 Edegem, Belgium

**Keywords:** T-cell receptor, TCR affinity, TCR avidity, TCR engineering, T-cell fitness, affinity-enhanced, cancer immunotherapy

## Abstract

Over the past decades, adoptive transfer of T cells has revolutionized cancer immunotherapy. In particular, T-cell receptor (TCR) engineering of T cells has marked important milestones in developing more precise and personalized cancer immunotherapies. However, to get the most benefit out of this approach, understanding the role that TCR affinity, avidity, and functional avidity play on how TCRs and T cells function in the context of tumor-associated antigen (TAA) recognition is vital to keep generating improved adoptive T-cell therapies. Aside from TCR-related parameters, other critical factors that govern T-cell activation are the effect of TCR co-receptors on TCR–peptide-major histocompatibility complex (pMHC) stabilization and TCR signaling, tumor epitope density, and TCR expression levels in TCR-engineered T cells. In this review, we describe the key aspects governing TCR specificity, T-cell activation, and how these concepts can be applied to cancer-specific TCR redirection of T cells.

## 1. Introduction to TCR Affinity, Avidity, and Functional Avidity

**Figure 1:**
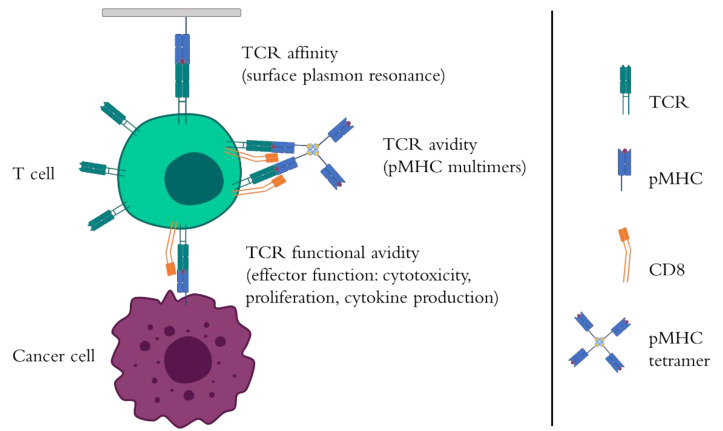
The interaction between the T-cell receptor (TCR) and the peptide-major histocompatibility complex (pMHC). T cells recognize tumor peptide epitopes via the pMHC. Different parameters affect the sensitivity that T cells, including T-cell receptor (TCR)-engineered T cells, will display against the pMHC. TCR affinity describes the strength of the interaction between a single TCR and pMHC. It is commonly measured using a technique named surface plasmon resonance. TCR avidity, on the other hand, reflects the contact of multiple TCRs and pMHCs. For this reason, multimers consisting of a number of pMHCs linked via streptavidin–biotin complexes to a fluorochrome are used to stain antigen-specific T cells and measure their TCR avidity. This parameter also takes into account the effect of T-cell co-receptors such as CD8 in the stabilization of TCR–pMHC binding. Closely related to TCR avidity, functional avidity shows the T-cell fitness to a target antigen in terms of its activation and effector functions, namely, T-cell proliferation, antitumor cytotoxicity, cytokine production, upregulation of activation markers, among others.

From tumor infiltrating lymphocytes to T-cell receptor (TCR) and chimeric antigen receptor (CAR) T-cell engineering, T cells have marked important milestones in cancer immunotherapy [1]. T cells recognize short peptide epitopes in the context of the major histocompatibility complex (MHC) thanks to their TCR. This receptor is a heterodimer of the immunoglobulin gene superfamily composed of two different alpha and beta polypeptides in conventional αβ T cells. The extracellular domain, which is involved in antigen recognition, comprises a variable region, a constant region, and a hinge, where a disulfide bridge is located to stabilize the interaction between the TCR chains. It continues into the transmembrane region and the intracellular domain, which interacts noncovalently with CD3 γ, δ, ε, and ζ proteins to form the TCR–CD3 complex. When a TCR correctly identifies the cognate peptide-MHC complex (pMHC), including the correct matching between MHC type and CD4/CD8 co-receptor, the TCR undergoes a series of conformational changes that lead to a first activation signal [2]. Three different TCR parameters are the major players governing this pMHC recognition and posterior T-cell activation, namely, TCR affinity, avidity, and functional avidity (Figure 1). TCR affinity is a key factor in controlling the sensitivity of the T cells towards the antigen and it is defined as the strength of the interaction between a single TCR and the pMHC ligand [3]. It is usually determined by the association (*k*_on_) and dissociation rates (*k*_off_) and represented as the equilibrium dissociation constant (*K*_D_) [3]. If TCR affinity relates to single receptors, TCR avidity measures the strength of multiple TCR–pMHC engagements and considers the effect of other molecules such as TCR co-receptors in the interaction, whereas functional avidity represents the T-cell fitness and activity at different concentrations of peptide epitope. The mean functional avidity is usually described as an EC50 concentration, representing the peptide dose at which a half-maximal activation of the T-cell population is reached [4]. Although physiological TCR affinities can range from 1 µM to 100 µM [5,6], several studies have marked the threshold of affinity for maximal T-cell activity, including antitumor T-cell responses, at 5–10 µM of peptide epitope [3,6,7,8]. In a comparative study of native TCRs with a TCR-like CAR, it was evidenced that the affinity of the antibody fragment in the TCR-like CAR was determinant to achieve better T-cell responses. In this study, and similarly to the ones performed by Zhong et al. [6], TCR affinity could not be improved above 5 µM, whereas TCR-like CARs would display an improved affinity threshold in the nM range [7]. Conversely, a comparison between conventional high-affinity single-chain TCRs and TCR-like CARs revealed that, although TCR-like CARs expression levels were higher, they were less sensitive in recognizing the ligand, which can be potential attributed to their signaling kinetics [9]. In a panel of TCRs with enhanced affinities within the physiological range against the cancer/testis antigen (CTA) New York esophageal squamous cell carcinoma 1 (NY-ESO-1), TCR-transduced T cells could respond to affinities above 5 µM, showing that affinity can limit the maximal activation of T cells [8]. This fact is likely caused by a reduced contribution of the TCR affinity to TCR avidity above the threshold [6]. Furthermore, a computational analysis from 12 phenotypic models of TCR–pMHC interactions showed that TCR affinity would not be a reliable marker for T-cell responses [10].

The TCR acquires its specificity in a maturation process that is based on somatic rearrangements of the variable (V), joining (J), and, only in the β chain, the diversity (D) TCR segments [11]. These rearrangements give rise to an almost infinite repertoire of TCRs with different specificities, including TCRs that recognize self-antigens, i.e., antigens naturally expressed in the human body. Many tumor-associated antigens (TAAs) targeted in adoptive T-cell therapies are self-antigens that can also be present in healthy tissues. Due to mechanisms of negative selection of auto-reactive lymphocytes, T-cell clones of high affinity against self-antigens are usually eliminated. Therefore, the frequency of high-affinity TCRs towards TAAs in circulating T-cells is low. In fact, natural cancer-specific TCRs usually promote an inferior T-cell response to physiological epitope densities, which would explain why tumors are able to avoid recognition by T cells [12]. On the contrary, TCRs with higher affinities and longer half-lives of TCR–pMHC binding kinetics commonly generate better T-cell responses because they can sense lower peptide epitope densities [12]. As the T-cell repertoire is edited and the affinity of circulating T cells against self-TAAs is usually low, in vitro affinity maturation is a potent tool to increase the ability of T cells to recognize low doses of peptide epitopes, which can even result in a 700-fold affinity increase [13]. However, it is important to highlight that affinity maturation may not always solve the problem of no recognition of low epitope densities, as it has been shown that affinity-matured TCRs with very high affinities improve the speed at which a T cell responds, but fail to respond to low density of pMHC [13]. This lack of recognition would be restored with lower TCR affinities that would lead to half-lives of more than 10 s, but half times ranging from 10^2^ to 10^3^ s would result in loss of sensitivity [13]. In a study analyzing the *k*_off_ rates of a library of low- and high-avidity cancer-specific T-cell clones after vaccination with different peptides, the dissociation rate was correlated with target recognition and Ca^2+^ mobilization [14]. More importantly, the affinity of the peptide used for the vaccine had a big impact on the avidity of the T-cell clones that were generated in patients after vaccination, with native and low-affinity peptides promoting the differentiation of cancer-specific T cells with higher avidity [14].

## 2. The Role of Epitope Density

T-cell activation is dependent on the binding kinetics of the TCR–pMHC, which in turn is influenced by the epitope density on the membrane of the tumor cell or antigen-presenting cell (APC) [15]. TAAs are processed intracellularly, bound to MHC molecules to form the pMHC, and presented on the cell membrane. The binding affinity between the tumor peptide and the MHC molecules has been linked with how T cells will respond. It appears that peptide-MHC affinities of 10 nM or higher are needed for tumor regression [16]. However, tumor peptide antigens are usually expressed in small amounts on the surface of tumor cells due to defects in their antigen processing and presentation machinery, such as downregulation of the levels of human leukocyte antigen (HLA) molecules [17]. In many cases, TAA levels are analyzed using mRNA-based techniques, which may misrepresent the actual pMHC numbers available for T cells [18]. In a peptidome analysis of predicted alternative splice forms, it was observed that peptides that are overabundant in cancer splice variants represent a minority of HLA class I epitopes in comparison to normal transcripts [19]. Moreover, hydrophilic amino acids were found to be more abundant in transcripts from cancer tissues, which may explain why cancer-specific peptides are less prone to be predicted as MHC epitopes [19]. Some studies have tried to understand the immunogenic profile of tumor cells in relation with the epitope density by using high-affinity soluble TCRs against immunodominant epitopes of CTAs NY-ESO-1 and L antigen family member 1 (LAGE-1), overexpressed TAAs, or differentiation-associated TAAs [20,21,22]. This technique has shown that naturally-processed TAA peptide epitopes are usually presented at ratios of 10 to 150 copies per cell [20]. These numbers would be sufficient for antigen-specific T cells as it has been demonstrated that one single TCR–pMHC interaction can induce T-cell activation in helper T cells [23]. This pMHC can engage with different TCR molecules and trigger T-cell activation after engaging with approximately 200 TCRs [24]. Moreover, three pMHC complexes are enough to promote cytotoxic T-cell killing [25]. However, more recent observations increased the number of pMHC ligands needed for correct T-cell activation to a minimum of 90 [26].

Although TCR affinity is directly correlated with the ability of the T cells of sensing lower densities of the antigen, TCR (functional) avidity predicts the capacity of a TCR-engineered T cell to induce a tumor-specific reaction when the number of pMHC is poor. Some evidence suggests that epitope density and not TCR affinity or avidity would play a major role in eliciting cancer-specific T-cell responses. In a non-Hodgkin B cell lymphoma mouse model, Segal and colleagues observed that avidity had not a major role in eliminating tumor burden [27]. Both high- and low-affinity TCRs successfully eradicated small tumors and were unable to respond against bigger tumors. Importantly, numbers of high-affinity T cells were reduced compared to low-affinity T cells, most probably due to the induction of apoptosis in the first group. T-cell fitness could be restored by changes in epitope density aiming to lower avidity from the side of the tumor. Similar observations have been described by Dougan and collaborators against the endogenous melanoma antigen tyrosinase-related protein 1 (TRP1) [28]. Another report argues that avidity is the major factor in eliminating leukemic cells in vivo, and not epitope density, the peptide-MHC affinity, nor the stability of the pMHC [29]. These findings support that there is a threshold of affinity and avidity above which further affinity enhancement or selection of supraphysiological avidities in T-cell clones would not translate in better in vivo responses. Hence, this challenges the way T-cell clones and TCRs are selected for preclinical and clinical testing. However, a study by Jaigirdar and colleagues indicated that high-avidity TCRs against the leukemia antigen Wilms’ tumor 1 (WT1) could not recognize naturally processed WT1 peptides [30]. These divergent studies highlight the complexity of TCR–pMHC interactions in the context of cancer recognition and the risk of oversimplifying the selection of T-cell clones or TCRs for TCR-engineering to the best TCR affinity or avidity.

## 3. The Role of TCR Co-Receptors

Once a TCR has engaged the corresponding pMHC, TCR co-receptors CD4 and CD8 bind to the invariant region of MHC class II and class I molecules, respectively. It is generally known that these co-receptors augment T-cell sensitivity and responses as the result of two main effects: (1) stabilization of weak interactions between the TCR and a cognate pMHC [31,32,33]; and (2) intracellular recruitment of the co-receptor-associated tyrosine kinase Lck to the vicinity of the TCR signaling complex, thereby enhancing the initiation of the TCR signaling cascade [34,35]. However, whereas numerous studies supported the role of CD8 in the latter effects, with TCR affinity threshold for CD8 dependence ranging from 60 to 120 µM [36], CD4 only acts to accelerate TCR-triggered signaling and not to stabilize TCR–pMHC interactions [37,38]. This ability is disputed by the extremely low affinity of CD4 for MHC molecules [39]. Nevertheless, the importance of co-receptor engagement in TCR binding to pMHC is illustrated by the fact that anti-CD4 and anti-CD8 antibodies can decrease or block and in the case of some antibody clones even enhance the extent to which the TCR interacts with pMHC [40,41]. This antibody blockade or enhancement is even more pronounced when a TCR binds with a low-affinity to pMHC [41]. Moreover, stabilization afforded by the extracellular domain of the CD8 co-receptor appears to be indispensable for enhanced activation of T cells with low-affinity TCRs, but not for T cells with high-affinity TCRs [42]. The CD8 co-receptor has been found to augment the binding efficiency at suboptimal TCR–pMHC affinities by altering both the association and dissociation rate of the TCR–pMHC interaction [43,44]. In addition, CD8 regulates the TCR sensitivity or triggering threshold by mobilizing TCR–pMHC class I complexes to membrane microdomains at a rate depending on the affinity of CD8 for MHC [44]. In contrast to the extracellular domain, the intracellular signaling domain of CD8 is critical for enhanced T-cell activation independent of the strength of the TCR [42]. Reduction of this CD8/Lck-dependent tyrosine kinase activity lowers the sensitivity of the TCR, and, therefore, impedes T-cell effector functions [45,46,47]. Based on these findings, the degree of dependency on CD8 to enhance T-cell functions differs depending on the affinity of its TCR for cognate pMHC. Furthermore, studies using pMHC multimers indicate the critical role of CD8 in antigen-specific TCR binding. Tetramers bearing a mutation in the CD8 binding site selectively bind to higher avidity T cells, but bind not to low avidity T cells [48]. Moreover, CD8 co-receptor engagement strengthens the avidity and stability of the interaction between T cells and their cognate multimers [48,49]. The aforementioned observations highlight how the presence or absence of TCR co-receptors impacts the interaction between T cells and cognate pMHC molecules. In addition, alterations in co-receptors expression levels or MHC binding capacity affect T-cell functionality as well. This is demonstrated by artificial mutations in the α3 domain of HLA-A2 that abrogate CD8 co-receptor binding, which resulted in inhibition of T cell-mediated specific lysis of target cells, without disturbing the TCR–pMHC interaction [50]. On the other hand, artificial altered HLA-A*68 molecules with enhanced CD8-binding ability induced an increase in T-cell proliferation and cytokine secretion [51]. The functional effects of a CD8–pMHC interaction are also underlined by the fact that IFN-γ secretion and CD107a surface expression of lower affinity pMHC-stimulated T cells could be achieved only in the presence of co-receptor engagement [43]. Lastly, CD8 synergy with low-affinity TCRs presents the issue of undesirable autoreactivity against self-peptides. However, T cells have the ability to reduce their functional avidity and thereby their autoreactive potential by downregulating CD8 membrane expression [52,53].

## 4. Selection of Cancer-Specific TCRs

A good starting point for searching cancer-specific TCR candidates is to isolate them from patients who have responded after treatment with peptide-based vaccines or dendritic cells (DCs) that have been engineered to express the full tumor antigen or pulsed with the target peptide (reviewed by [54]). The application of peptide-based or antigen mRNA-based cancer vaccines using DCs focuses on the increment of epitope density on the surface of antigen-presenting cells to boost the immune system against one or multiple TAAs (reviewed by [55,56]). When patient cells are not available, using donor material is another alternative. High-avidity T-cell clones from a naïve repertoire can be isolated using autologous peptide-loaded monocyte-derived DCs, followed by subsequent restimulation with peptide-loaded peripheral blood mononuclear cells (PBMC) [57]. Although this can be difficult to achieve due to the scarcity of highly reactive clones against self-antigens. Another source of tumor-reactive T-cell clones is allogeneic material. In this case, cells from mismatched donors are used aiming to achieve alloreactive T cells specific towards the full pMHC rather than the peptide alone [58,59,60]. Alternatively, transgenic mice that have been vaccinated with the target peptide represent a source of murine TCRs usually defined by a high affinity towards the ligand [61,62,63]. However, one drawback of this strategy is that allogeneic TCRs can show epitope promiscuity and could potentially cause off-target reactivities [58]. To improve the specificity and the affinity of the TCR candidates, viral antigens can be used from virus-associated malignancies (reviewed by [64]), but the usage of reactive T cells against these epitopes will be limited to a certain number of patients. T-cell clones reactive to tumor neoantigens are gaining momentum since the latter are truly cancer epitopes that are not found in healthy tissues [65]. These neoantigen-specific T cells provide a source of highly specific tumor-reactive TCRs for genetic transfer [66,67]. Nevertheless, this approach presents some challenges related to the correct identification of candidate neoepitopes, and thus to that of neoantigen-specific T-cell clonotypes, as well as other challenges related to the heterogeneity of tumor mutations and the epitope density of these antigens [68].

Regardless of their origin, the selected TCR candidates should undergo further testing to ensure their specificity and efficacy, by both binding assays with pMHC multimers and functional assays [69] (Figure 1). This is especially important due to the weaker binding strength of TCR against self-antigens versus, for example, viral antigens [69]. This correlation between TCR affinity and T-cell immune responses is clearly evidenced by the difference in how T cells engineered with virus (higher affinity) or cancer-specific (lower affinity) TCRs respond [69]. In addition, high-affinity TCRs tend to rely less on the effect of CD8 co-receptor binding than low affinity TCRs [69]. The use of pMHC multimers has been extensively used as the first method of choice to analyze TCR avidity, especially for CD8-positive T cells, as the detection of antigen-specific CD4 T cells using pMHC class II multimers is still challenging [70,71]. However, as described before, pMHC multimers do not provide information on functional avidity or may not even identify important antigen-specific TCR repertoires [72]. For this purpose, Morimoto and colleagues developed a TCR-deficient CD8-positive Jurkat-derived cell line to rapidly and uniformly evaluate the functional avidity of cloned TCRs [73]. This cell line, called 2D3, provides a way to homogenize/standardize the measurement of T-cell functional avidity. It is provided with a nuclear factor of activated T-cells (NFAT)-driven enhanced green fluorescent protein (EGFP) reporter gene so that TCR activation can be linked to EGFP expression [73]. One of the advantages of this cell line is that it can be easily genetically modified with DNA or mRNA encoding the TCR using any type of engineering method [73,74]. Rosskopf and colleagues went further by adding three fluorescent proteins: EGFP, cyan fluorescent Protein (CFP), and mCherry to another Jurkat-derived cell line. With this triple parameter reporter platform, up to three transcription factors—NFAT, nuclear factor kappa-light-chain-enhancer of activated B cells (NF-κB), and activator protein 1 (AP-1), which play key roles in T-cell activation—can be analyzed at the same time to evaluate TCR function [75]. CD137 is an activation marker upregulated 24 h after stimulation of CD8 T cells and can be used as an enrichment marker for high-avidity T-cell clones of different expanded T-cell subsets from a naïve repertoire [76]. One of the greatest challenges of selecting affinity-optimized TCRs is to diminish the risk of on-target or off-target cross-reactivities. Border et al. described a scanning method with which effective TCRs could be identified while pinpointing those that could be potentially dangerous TCRs [77]. This scanning method is based on a first selection of natural TCRs based on affinity and functional avidity followed by the affinity enhancement of those TCRs and further affinity and functional characterization. The final candidates are then compared by using an X-scan, a system in which all the residues of the peptide of interest are mutated into every amino acid possible. This extensive screening ensures that the candidates will not recognize other potential peptides but the target one.

Our group has also highlighted the importance of selecting the correct APC to correctly analyze TCR avidity [4]. To analyze TCR avidity and to predict the sensitivity of cancer-specific TCR-engineered T cells, APCs are pulsed with different concentrations of peptide antigens, usually in the micromolar range. In particular, the T2 cell line, a T cell-B cell hybridoma, has become the gold standard in this type of assay. This cell line presents a deficiency in transporter associated with antigen processing (TAP) proteins, which leads to the presence of “empty” HLA molecules on the cell surface. Although this feature is desirable in peptide-pulsing assays, the overabundance of the pulsed peptide above physiological levels compared to those of naturally-processed TAA peptides may lead to misrepresentation of the TCR avidity. In fact, when peptide-pulsing assays are commonly performed using micromolar amounts of peptide [74,78], T2 cells would need to be pulsed with low nanomolar concentrations to resemble physiological amounts of epitopes [21]. Certainly, other cell lines and assays to investigate tumor killing, cytokine production (important for adverse effects related to cytokine storms), and, in general, any other indicator of T-cell fitness and specificity for antitumor responses are possible.

## 5. Improvement of TCR-Engineered T-Cell Antitumor Responses

**Figure 2:**
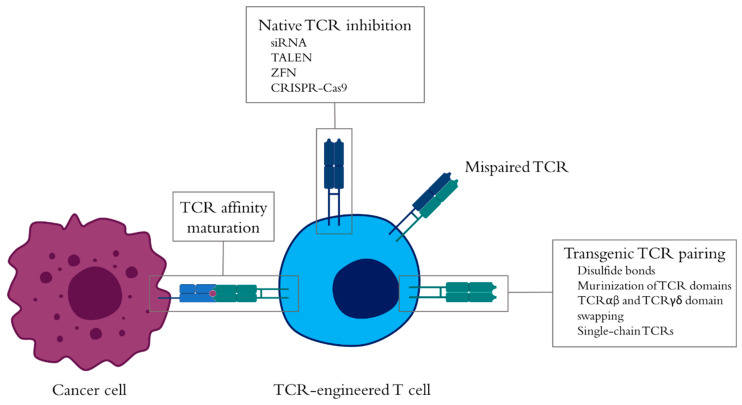
Enhancement of tumor-specific T-cell receptor (TCR)-engineered T cells. The antitumor functionality of TCR-engineered T cells can be leveraged by improving the affinity of the TCR–peptide-major histocompatibility complex (pMHC) interaction via TCR affinity maturation processes, such as phage display or the substitution of key amino acids in the complementarity-determining regions (CDRs) of the TCR. On another note, the presence of native and transgenic TCRs can lead to the mispairing of their TCR chains that reduce the levels of transgenic TCR on the surface of the T cells. To overcome this problem, the presence of native TCRs can be either downregulated by silencing RNAs targeting the TCR constant sequences in mRNA transcripts or completely abrogated with tools such as zinc-finger nucleases (ZFNs)**,** transcription activator-like effector nucleases (TALENs), or the clustered regularly interspaced short palindromic repeats (CRISPR)-Cas9 system. These techniques can be combined with the improvement of TCR pairing by addition of disulfide bonds, the murinization of TCR αβ constant domains, or the use of TCR γδ domains in the TCR αβ. Finally, systems in which the two TCR chains are transformed into one single TCR chain can also ensure that mispairing with the native TCRs does not occur without the need to abolish its expression.

Despite some divergences in the correlation between TCR affinity and T-cell activity, the selection of high-affinity TCRs or the affinity enhancement of low-affinity TCRs constitutes a mean to improve antitumor responses (Figure 2). Different techniques are employed for TCR affinity maturation, including the phage display system—which can achieve TCR affinities in the picomolar range [3,79,80], the yeast TCR display system [81], a mammalian retroviral display system coupled with an alanine-scanning approach to identify key amino acid residues [82], the substitution of key amino acids in the TCR complementarity-determining regions (CDRs) [83,84,85], or the use of somatic hypermutation [86]. On another note, enhancement of transgenic TCR dimerization and TCR availability on the surface of the T cells represents a way to improve TCR avidity and, hence, T-cell functionality [36]. One of the pitfalls in TCR engineering is the low expression of transgenic TCRs due to mispairing with native TCRs, which in turn can give rise to deleterious reactivities, and competition for the TCR complex machinery [87,88,89,90]. Multiple techniques have been developed over the years to solve this problem, focusing on different aspects of the TCR machinery (Figure 2). A way to improve the amount of transgenic TCRs available on the cell surface, while reducing the presence of the native TCRs is by silencing the native TCR sequences using short hairpin RNAs either included in the same vector where the transgenic TCR is located [91,92,93,94] or by transfection of silencing RNAs (siRNAs). In both cases, the siRNAs are directed against the constant regions of the TCR chains to target multiple native TCR sequences at a time. The complete removal of the native TCR can be achieved by techniques such as zinc-finger nucleases (ZFNs) [95], transcription activator-like effector nucleases (TALENs) [96,97,98], or, more recently, the clustered regularly interspaced short palindromic repeats (CRISPR)-Cas9 system [97,98,99,100]. Although native TCR inhibition is a simple way to reduce TCR mispairing, other strategies tackle the stability of the transgenic TCR and, by doing so, they reduce TCR mispairing (Figure 2). Thus, TCRs for genetic engineering of T cells have been modified with extra disulfide bonds [101,102,103], which has recently been employed in high-affinity soluble TCRs [104]. This is achieved by introducing cysteines in both the TCR alpha and beta chains. Alternatively, the constant domains of human TCR chains can be substituted for either murine αβ or human γδ TCRs. With this strategy, the constant regions of the αβ TCR chains are swapped to produce chimeric TCRs that retain their antitumor functionality [105,106,107,108,109]. Despite enhanced TCR antitumor functionality, the presence of xenogeneic material may result in immunogenicity that could hinder the effect of the cells. This issue can be addressed by substituting key residues in the constant region of the TCR with those of murine origin [110]. Furthermore, while this strategy still produces mispaired TCRs, these are unable to bind to CD3 rendering them ineffective [105]. However, with these strategies, mispairing can still occur. To largely avoid incorrect pairing, single chain TCRs are based on the fusion of the variable regions of the TCR alpha and beta chains connected with a linker [111]. This structure is then joined to the TCR beta constant region to form the single chain TCR, whereas the constant TCR alpha is added separately to allow the recruitment of the CD3 complex. Similar to a full TCR, the addition of an extra disulfide bond in the variable region strengthens the stability of the molecule and even improves the functional activity of engineered cells [111]. These alterations of either the pool of native TCRs or the structure of the transgenic TCR can of course be combinable to further increase TCR avidity and promote better T-cell responses.

## 6. Clinical Impact of TCR Affinity and Avidity in Cancer-Specific TCR-Engineered T Cells

Cancer-specific TCR-engineered T cells have been used in the clinic for more than a decade [112], concurrently with the idea that TCR affinity and avidity would have a major role in successfully eliminating cancer cells [113]. Affinity-matured TCR-engineered T cells have been successful in inducing clinical responses in tumors expressing the melanoma antigen recognized by T cells (MART-1) [114,115,116], glycoprotein 100 (gp100) [114], WT1 protein [117], carcinoembryonic antigen (CEA) [118], NY-ESO-1 [119,120,121,122,123,124], LAGE-1 [124], or the melanoma-associated antigen A (MAGE-A) family [125,126,127,128]. Antitumor affinity-enhanced TCRs, although they increase the recognition of tumor cells with low epitope density, they also increase the risk of cross-reactivity with antigens from normal tissues. Off-target recognition and cross-reactivity has been demonstrated in clinical trials using affinity-enhanced TCRs [118,125,127,128]. T cells engineered with an affinity-enhanced HLA-A*02-restricted TCR isolated from immunized mice with CEA peptide led to severe transient colitis [118]; whereas an affinity-enhanced HLA-A*02-restricted MAGE-A3/A9/A12-specific TCR derived from MAGE-A3-vaccinated transgenic mice caused neurotoxicity due to the recognition of MAGE-A12 expressed by brain cells [125]. Another high-affinity HLA-A*01-restricted MAGE-A3-specific TCR, developed against myeloma and melanoma, led to cardiogenic shock and ultimate death of the first two treated patients [127]. Preclinical studies showed no predicted off-target reactivities [128]; however, T cells engineered with this TCR caused severe cardiac tissue damage in patients due to the recognition of a striated muscle-specific titin-derived peptide [127,128]. Although lethal adverse events can also occur with TCRs that have not undergone affinity enhancement [129], this study showed the risks of using affinity-enhanced TCRs without extensive prior testing of cross-reactivities. To address this issue, Sanderson and colleagues developed an in vitro extensive preclinical testing protocol to evaluate the safety and efficacy of an affinity-enhanced MAGE-A4-specific TCR by using a wide range of testing material, including human tumor cell lines, primary tumor material, and panels of EBV-transformed B-lymphoblastic cell lines (B-LCLs) expressing multiple HLA alleles and molecular analysis [130]. After undergoing this testing procedure, Sanderson and colleagues obtained an affinity-enhanced TCR candidate with a safe clinical profile to test in clinical trials (NCT03132922, NCT04044768). Another issue involving affinity-enhanced TCRs revolves around the constant tonic signaling by recognition of the HLA molecules. Although this problem initially may not put the lives of patients at risk, it impairs the functional activity of the engineered T cells due to TCR–CD3 downregulation and upregulation of inhibitory receptors [131]. On the bright side, this constant TCR activation may be prevented by fine-tuning the affinity of the TCR [131].

TCR mispairing between the endogenous and the transgenic TCR chains, although not limited to high-affinity TCRs, is a concern to be taken into consideration for the safety of adoptive TCR-engineered T cell therapies [88,89]. Even though adverse events caused by neoreactivities linked to TCR mispairing have not been reported so far, it is an underlying issue that can be solved by disruption of the endogenous TCR using multiple techniques (Figure 2), some of which have already been tested in the clinic with positive results [117,132,133]. In particular, the CRISPR-Cas9 system has revolutionized the way cells are genetically engineered for the treatment of cancer due to its simplicity, fidelity, and versatility [134]. Very recently, this method has been employed in refractory cancer patients to modify T cells with a cancer-specific TCR while suppressing the endogenous TCR chains and the negative immune checkpoint programmed cell death protein 1 (PD-1) genes in a multiplex system [132].

In patients where it is difficult to isolate cancer-specific TCRs, T cells from healthy donors can be a good alternative [135]. One of the advantages of this option is that an indefinite number of donors, whose T cell numbers are not compromised, can be screened until achieving the best high-affinity TCRs. However, the HLA repertoire of patients and donors should be matched to prevent alloreactivities from the endogenous donor TCR [135]. Another issue of this strategy is the potential off-target reactivities also caused by the donor TCR, which can be prevented using the same techniques employed to minimize TCR mispairing. Due to the potential severe toxicities of TCRs derived from cytotoxic CD8 T cells, high-affinity TCRs obtained from regulatory T cells (Tregs) [136] or helper CD4 T cells [137,138] represent an alternative source of cancer-specific TCRs. Although the use of Treg-derived TCRs raise concerns regarding the possibility of redirection of engineered helper CD4 T cells into Tregs in vivo, this was not observed in patients so far and instead induced tumor regression in metastatic cancer patients [139].

## 7. Conclusions and Future Perspectives

The delicate interconnection between TCR affinity, avidity, the co-receptors, and the epitope density highlights the importance of finding a balance between increased TCR affinity or avidity to sense low epitope densities and supraphysiological T-cell activity to avoid potentially dangerous cross-reactivities. In this direction, new ways to produce TCRs with fine-tuned affinities [140], de novo generation of tumor-specific TCRs [141] and the selection of neoantigens [142,143] or TAP-independent antigens [143,144] as epitopes for tumor targeting will be beneficial to produce more effective and safer TCR-modified T cells. The future of TCR therapies is increasingly becoming not limited to conventional T cells, as non-conventional lymphocytes such as γδ T cells, and their TCRs, and natural killer cells are being explored in pre-clinical and clinical settings [145,146,147,148]. These cell types bypass concerns related to TCR mispairing and cross-reactivities, while having an intrinsic antitumor activity. They also offer the possibility of producing off-the-self allogeneic products due to their lack of graft-versus-host complications. Additionally, combinatorial approaches to improve T-cell activity with cytokines or immune checkpoints inhibitors may eliminate the need to produce TCRs with supraphysiological affinities that may cause severe adverse effects [132,149,150,151]. In summary, the complexity of the TCR–pMHC interactions, and thus that of T cell–tumor cell interactions, will require TCR genetic engineering to take a holistic approach to develop more precise and effective adoptive T-cell cancer therapies.
